# Supplementary material for: The thioredoxin-1 system is essential for fueling DNA synthesis during T-cell metabolic reprogramming and proliferation
Source: Nat Commun. 2018 May 10;9:1851. doi: 10.1038/s41467-018-04274-w (PMC5945637; doi:10.1038/s41467-018-04274-w)
Supplement: Supplementary file 1 — Supplementary Information [file 41467_2018_4274_MOESM1_ESM.pdf]

Supplementary Information

The thioredoxin-1 system is essential for fueling DNA synthesis during T cell metabolic reprogramming and proliferation

Muri et al.

Supplementary Figures

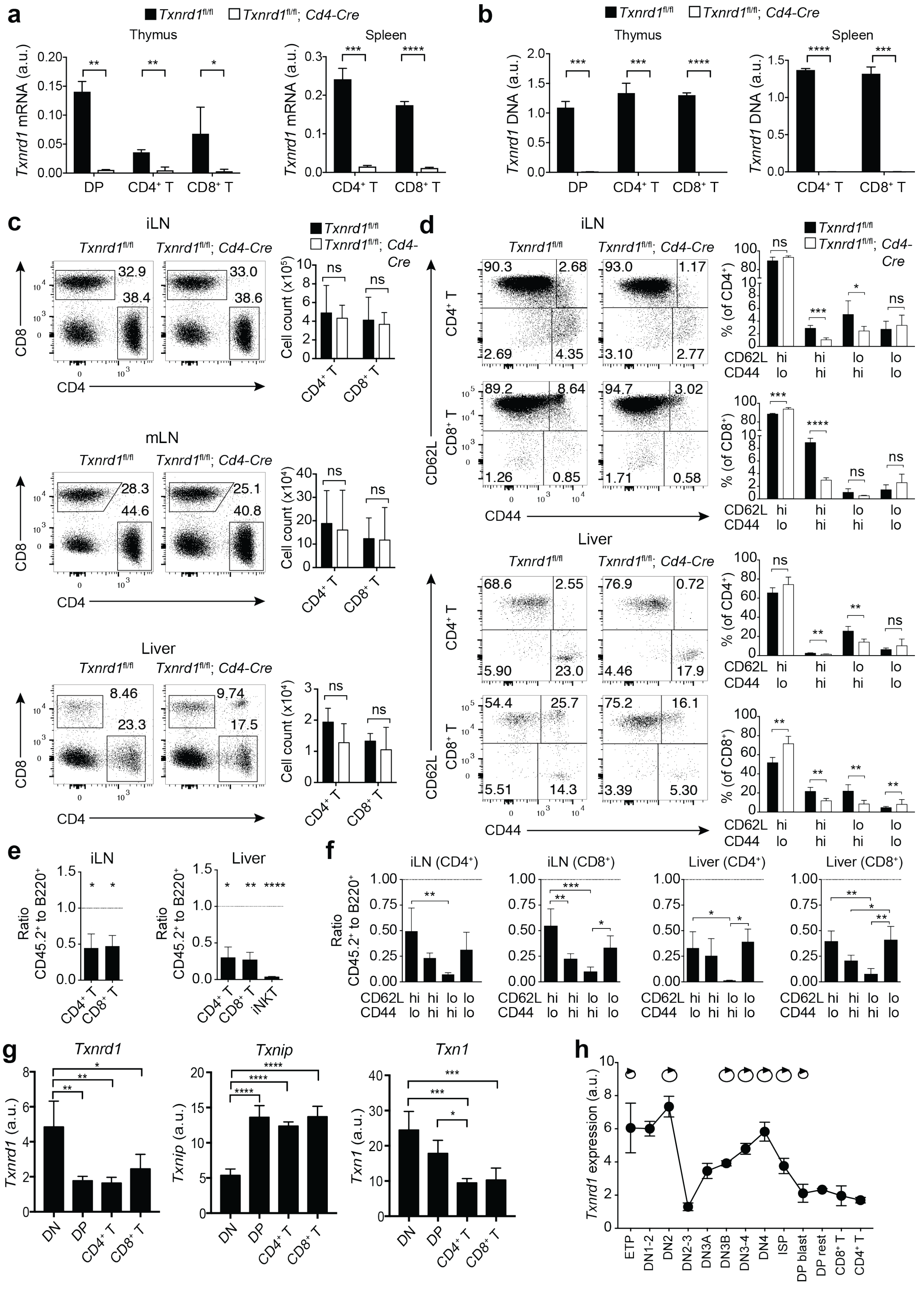

Supplementary Figure 1. *Txnrd1* is required for the generation of activated/memory T cells. (**a, b**) Analysis of *Txnrd1* mRNA (**a**) and genomic *Txnrd1* DNA (**b**) in DP and SP thymocytes (left), and splenic T cells (right) determined by RT-PCR (n=4). (**c, d**) Numbers and percentages of the indicated T cell populations were analyzed in naïve *Txnrd1$^{fl/fl}$;Cd4-Cre* and *Txnrd1$^{fl/fl}$* littermate control mice. Representative FACS plots (left) and quantification (right) are shown (n=4-5). (**c**) $CD4^+$ $TCR\beta^+$ ($CD4^+$ T) and $CD8^+$ $TCR\beta^+$ ($CD8^+$ T) T cells in inguinal lymph nodes (iLN; top), mesenteric LNs (mLN; middle) and livers (bottom). (**d**) Expression of CD62L and CD44 in iLN (top) and liver (bottom) T cells. (**e, f**) Lethally irradiated WT mice were reconstituted with a 1:1 mixture of WT and *Txnrd1$^{fl/fl}$;Cd4-Cre* bone marrow expressing the congenic markers CD45.1 and CD45.2, respectively. After reconstitution, the indicated T cell populations in iLN and liver from *Txnrd1$^{fl/fl}$;Cd4-Cre* donor mice were normalized to non-Cre expressing $CD45.2^+CD19^+$ B cells. A value below 1 indicates a reduced contribution of *Txnrd1*-deleted T cells to the indicated population compared to WT cells (n=4). (**g**) Expression levels of *Txnrd1, Txnip* and *Txn1* determined by RT-PCR for sorted DN ($CD4^-CD8^-TCR\gamma\delta^-$), DP ($CD4^+CD8^+$), $CD4^+$ ($CD4^+CD8^-TCR\beta^+$) and $CD8^+$ ($CD8^+CD4^-TCR\beta^+$) populations from WT mice (n=4). (**h**) Expression levels of *Txnrd1* determined by RT-PCR for the indicated FACS-sorted thymocyte populations from WT mice. Circular arrows indicate proliferating populations (n=4). Bar graphs show mean + standard deviation. Data are representative of two (**a, b, e-h**) and three (**c, d**) independent experiments. Student's *t* test (two-tailed, unpaired) was used to compare *Txnrd1$^{fl/fl}$* and *Txnrd1$^{fl/fl}$;Cd4-Cre* groups (**a-d**): *, P ≤ 0.05; **, P ≤ 0.01; ***, P ≤ 0.001; ****, P ≤ 0.0001; ns, not significant. One-sample t test with a hypothetical value of 1 was used in (**e**): *, P ≤ 0.0332; **, P ≤ 0.0021; ****, P ≤ 0.0001. One-way AVOVA adjusted by Tukey's multiple comparison test was used in (**f, g**): *, P ≤ 0.05; **, P ≤ 0.01; ***, P ≤ 0.001.

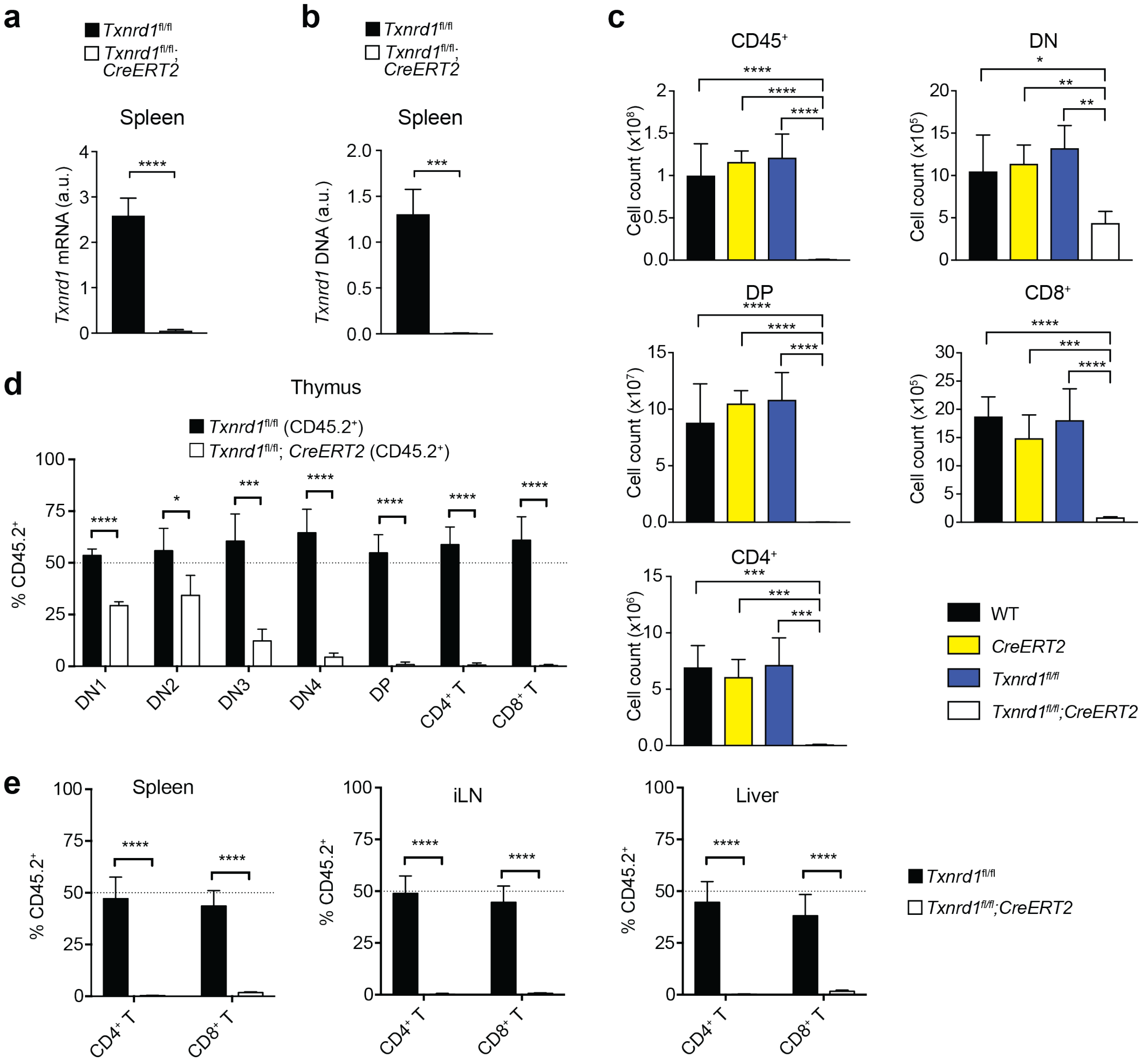
a
Txnrd1fl/fl
Txnrd1fl/fl; CreERT2
Spleen
Txnrd1 mRNA (a.u.)
****
b
Txnrd1fl/fl
Txnrd1fl/fl; CreERT2
Spleen
Txnrd1 DNA (a.u.)
***
c
CD45+
DN
DP
CD8+
CD4+
Cell count (x10^8)
Cell count (x10^5)
Cell count (x10^7)
Cell count (x10^6)
WT
CreERT2
Txnrd1fl/fl
Txnrd1fl/fl;CreERT2
d
Thymus
Txnrd1fl/fl (CD45.2+)
Txnrd1fl/fl; CreERT2 (CD45.2+)
% CD45.2+
DN1
DN2
DN3
DN4
DP
CD4+ T
CD8+ T
e
Spleen
iLN
Liver
% CD45.2+
CD4+ T
CD8+ T
Txnrd1fl/fl
Txnrd1fl/fl;CreERT2

Supplementary Figure 2. *Txnrd1* is critically required for thymic T cell development. (**a, b**) Analysis of *Txnrd1* mRNA (**a**) and genomic *Txnrd1* DNA (**b**) in splenic T cells determined by RT-PCR (n=4). (**c**) Total numbers of the indicated thymic cell populations from WT, *Cre-ERT2*, *Txnrd1*$^{fl/fl}$ and *Txnrd1*$^{fl/fl}$*;Cre-ERT2* mice (n=4-5). (**d, e**) Lethally irradiated WT mice ($CD45.1^+$) were reconstituted with a mixture (1:1) of WT ($CD45.1^+CD45.2^+$) and TAM-treated *Txnrd1*$^{fl/fl}$*;Cre-ERT2* ($CD45.2^+$) bone marrow (or *Txnrd1*$^{fl/fl}$ as control). After reconstitution, the percentage of donor $CD45.2^+$ cells among the indicated cell populations was determined by flow cytometry (n=4-5). Shown are the percentages of the indicated thymic T cell populations (**d**) and of $CD4^+$ and $CD8^+$ T cells in spleen, inguinal LN and liver (**e**). Bar graphs show mean + standard deviation. Data are representative of two independent experiments. Student's *t* test (two-tailed, unpaired) was used to compare *Txnrd1*$^{fl/fl}$ and *Txnrd1*$^{fl/fl}$*;Cre-ERT2* groups (**a, b, d, e**): **, P ≤ 0.01; ***, P ≤ 0.001; ****, P ≤ 0.0001. One-way AVOVA adjusted by Tukey's multiple comparison test was used in (**c**): *, P ≤ 0.05; **, P ≤ 0.01; ***, P ≤ 0.001.

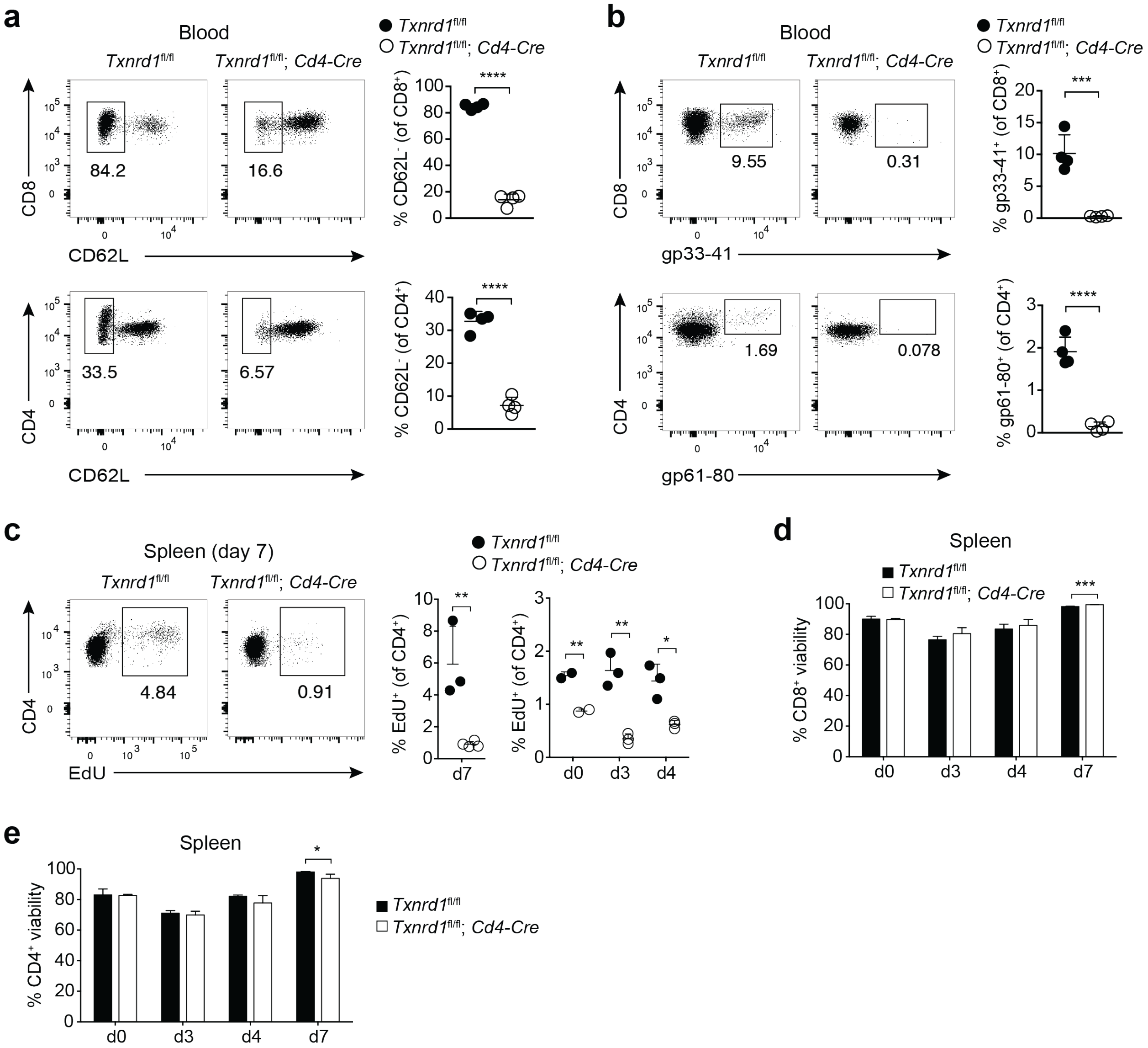

a
Blood
Txnrd1fl/fl
Txnrd1fl/fl; Cd4-Cre
CD8
CD62L
84.2
16.6
% CD62L- (of CD8+)
****
CD4
33.5
6.57
% CD62L- (of CD4+)
b
Blood
gp33-41
9.55
0.31
% gp33-41+ (of CD8+)
***
gp61-80
1.69
0.078
% gp61-80+ (of CD4+)
c
Spleen (day 7)
EdU
4.84
0.91
% EdU+ (of CD4+)
d7
d0
d3
d4
d
Spleen
% CD8+ viability
d7
e
Spleen
% CD4+ viability

Supplementary Figure 3. T cells require *Txnrd1* for efficient expansion during LCMV infection. *Txnrd1$^{fl/fl}$* and *Txnrd1$^{fl/fl}$;Cd4-Cre* mice were infected with 200 focus-forming units (FFU) LCMV WE i.v., and T cells in blood were analyzed after 3, 4 and 7 days of infection. (**a-b**) On day 7 of infection, activation of $CD4^+$ and $CD8^+$ T cells in blood was assessed by down-regulation of CD62L (**a**), and virus-specific T cells were stained with gp_{33-41} and gp_{61-80} tetramers (**b**). (**c**) Proliferation of splenic $CD4^+$ T cells was assessed by EdU incorporation. Representative FACS plots (pre-gated on $CD4^+$) and percentages of EdU^+ cells are shown on the left and right side, respectively. (**d, e**) Cell death was assessed by staining with Annexin-V and 7-AAD, and percentages of alive (Annexin-V^- 7-AAD^-) $CD8^+$ (**d**) and $CD4^+$ (**e**) T cells are shown (n=2-4). Means + standard deviations are shown. Data are representative of two independent experiments. Student's *t* test (two-tailed, unpaired) was used to compare *Txnrd1$^{fl/fl}$* and *Txnrd1$^{fl/fl}$;Cd4-Cre* groups (**a-e**): *, P ≤ 0.05; **, P ≤ 0.01; ***, P ≤ 0.001; ****, P ≤ 0.0001.

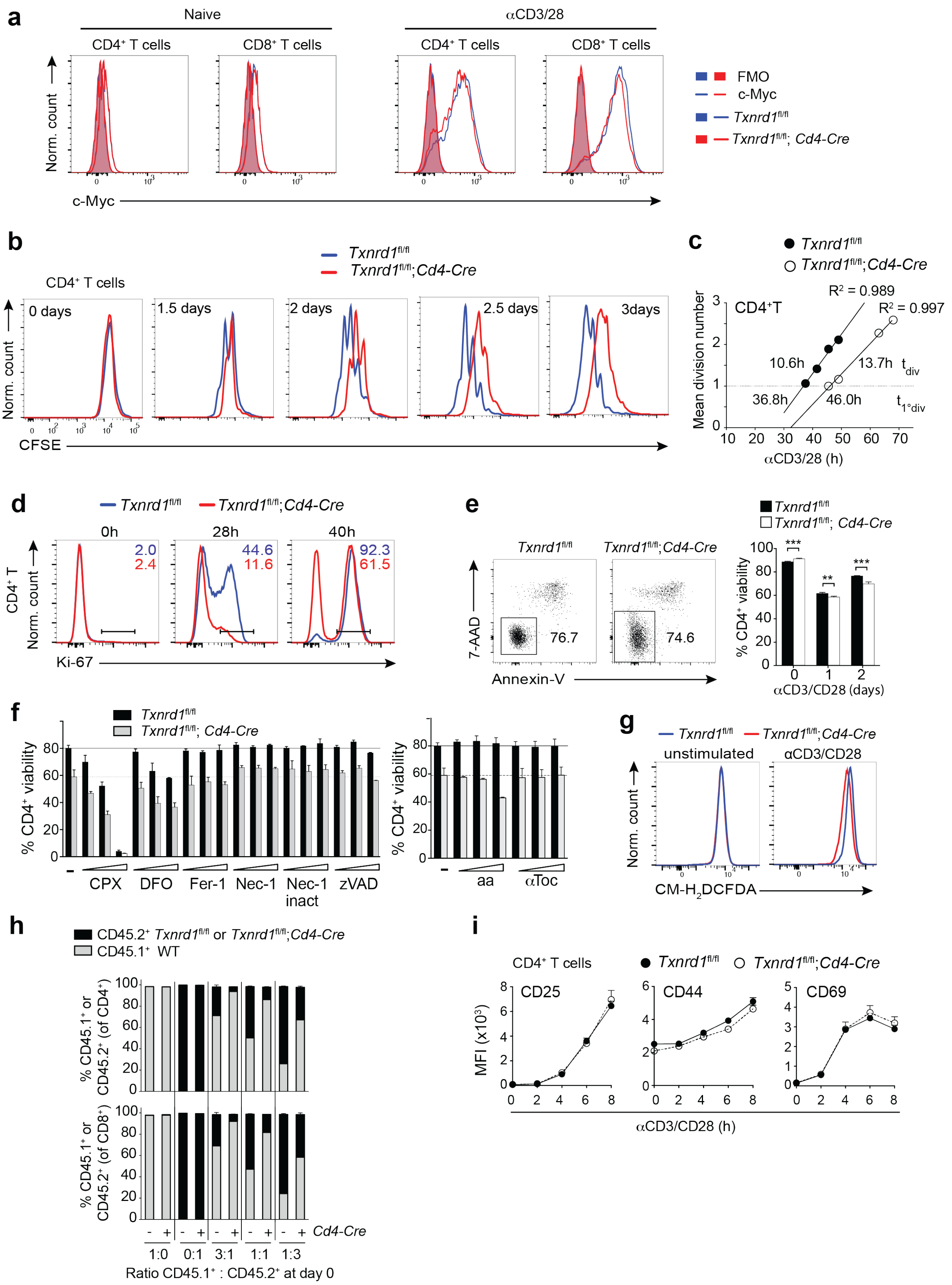
a
Naive
αCD3/28
CD4+ T cells
CD8+ T cells
Norm. count
c-Myc
FMO
c-Myc
Txnrd1fl/fl
Txnrd1fl/fl; Cd4-Cre
b
CD4+ T cells
Txnrd1fl/fl
Txnrd1fl/fl;Cd4-Cre
0 days
1.5 days
2 days
2.5 days
3days
Norm. count
CFSE
c
Txnrd1fl/fl
Txnrd1fl/fl;Cd4-Cre
CD4+T
R2 = 0.989
R2 = 0.997
10.6h
13.7h
tdiv
36.8h
46.0h
t1°div
Mean division number
αCD3/28 (h)
d
Txnrd1fl/fl
Txnrd1fl/fl;Cd4-Cre
0h
28h
40h
2.0
2.4
44.6
11.6
92.3
61.5
CD4+ T
Norm. count
Ki-67
e
Txnrd1fl/fl
Txnrd1fl/fl;Cd4-Cre
76.7
74.6
7-AAD
Annexin-V
Txnrd1fl/fl
Txnrd1fl/fl; Cd4-Cre
***
**
***
% CD4+ viability
αCD3/CD28 (days)
f
Txnrd1fl/fl
Txnrd1fl/fl; Cd4-Cre
% CD4+ viability
CPX
DFO
Fer-1
Nec-1
Nec-1 inact
zVAD
aa
αToc
g
Txnrd1fl/fl
Txnrd1fl/fl;Cd4-Cre
unstimulated
αCD3/CD28
Norm. count
CM-H2DCFDA
h
CD45.2+ Txnrd1fl/fl or Txnrd1fl/fl;Cd4-Cre
CD45.1+ WT
% CD45.1+ or CD45.2+ (of CD4+)
% CD45.1+ or CD45.2+ (of CD8+)
Cd4-Cre
1:0
0:1
3:1
1:1
1:3
Ratio CD45.1+ : CD45.2+ at day 0
i
CD4+ T cells
Txnrd1fl/fl
Txnrd1fl/fl;Cd4-Cre
CD25
CD44
CD69
MFI (x103)
αCD3/CD28 (h)

Supplementary Figure 4. *Txnrd1*-deficient T cells undergo longer cell cycling. Magnetically-sorted T cells from spleen and peripheral LNs of $Txnrd1^{fl/fl}$ and $Txnrd1^{fl/fl}$*;Cd4-Cre* mice were stimulated in αCD3/CD28-coated plates followed by flow cytometry analysis. (**a**) c-Myc protein expression level after 20 hours of T cells stimulation. (**b**) CFSE dilution of $CD4^+$ T cells at the indicated times of stimulation. (**c**) CFSE-dilution profiles were used to calculate mean division time ($t_{division}$; reciprocal of the slope) and time to first division ($t_{1°division}$; intersection with mean division number y = 1) with the *precursor cohort method*. (**d**) $CD4^+$ T cells leaving G_0 phase and entering cell cycle were detected by staining for Ki-67 at the indicated times of stimulation. Numbers in the plots indicate average percentage of Ki-67^+ cells from triplicate samples. (**e**) $CD4^+$ T cell death during αCD3/CD28 stimulation was assessed by staining cells with Annexin-V and 7-AAD. Representative FACS plots of cells stimulated for 1 day as well as quantification of alive (Annexin-V^- 7-AAD^-) cells at the indicated times of stimulation (n=4). (**f**) Analysis of $CD4^+$ T cell survival upon incubation with the indicated cell death inhibitors and antioxidants during 24 hours of αCD3/CD28 stimulation. Results for treatment with the ferroptosis inhibitors ciclopirox olamine (CPX; 8, 63, 500 nM), Deferoxamine (DFO; 1, 10, 50 μM) and Ferrostatin-1 (Fer-1; 0.5, 2, 8 μM); the necroptosis inhibitor Necrostatin-1 (Nec-1; 1, 4, 20 μM) and the apoptosis inhibitor z-VAD-fmk (zVAD; 2, 12, 60 μM) are shown in the left panel. Survival of T cells after addition of the antioxidants ascorbic acid (1, 6, 30 μM) and α-Tocopherol (αToc; 3, 16, 80 μM) is shown in the right panel (n=2). (**g**) Representative FACS plots of ROS levels in unstimulated (left) and αCD3/CD28-stimulated T cells (right), stained with CM-H_2DCFDA. (**h**) T cells from WT ($CD45.1^+$) and from $Txnrd1^{fl/fl}$*;Cd4-Cre* mice ($CD45.2^+$; or $Txnrd1^{fl/fl}$ as control) were mixed at the indicated ratios (1:0, 0:1, 3:1, 1:1, 1:3) and stimulated for 3 days. Percentages of $CD45.1^+$ (gray) and $CD45.2^+$ (black) for $CD4^+$ (top) and $CD8^+$ (bottom) are shown (n=3). (**i**) Expression of indicated activation markers in $CD4^+$ T cells measured by flow cytometry during the first 8h of stimulation (n=3). Bar graphs show mean + standard deviation. Data are representative of two (**a, e-i**), five (**b**), and three (**c, d**) independent experiments. Student's *t* test (two-tailed, unpaired) was used in (**e**): **, $P \leq 0.01$; ***, $P \leq 0.001$.

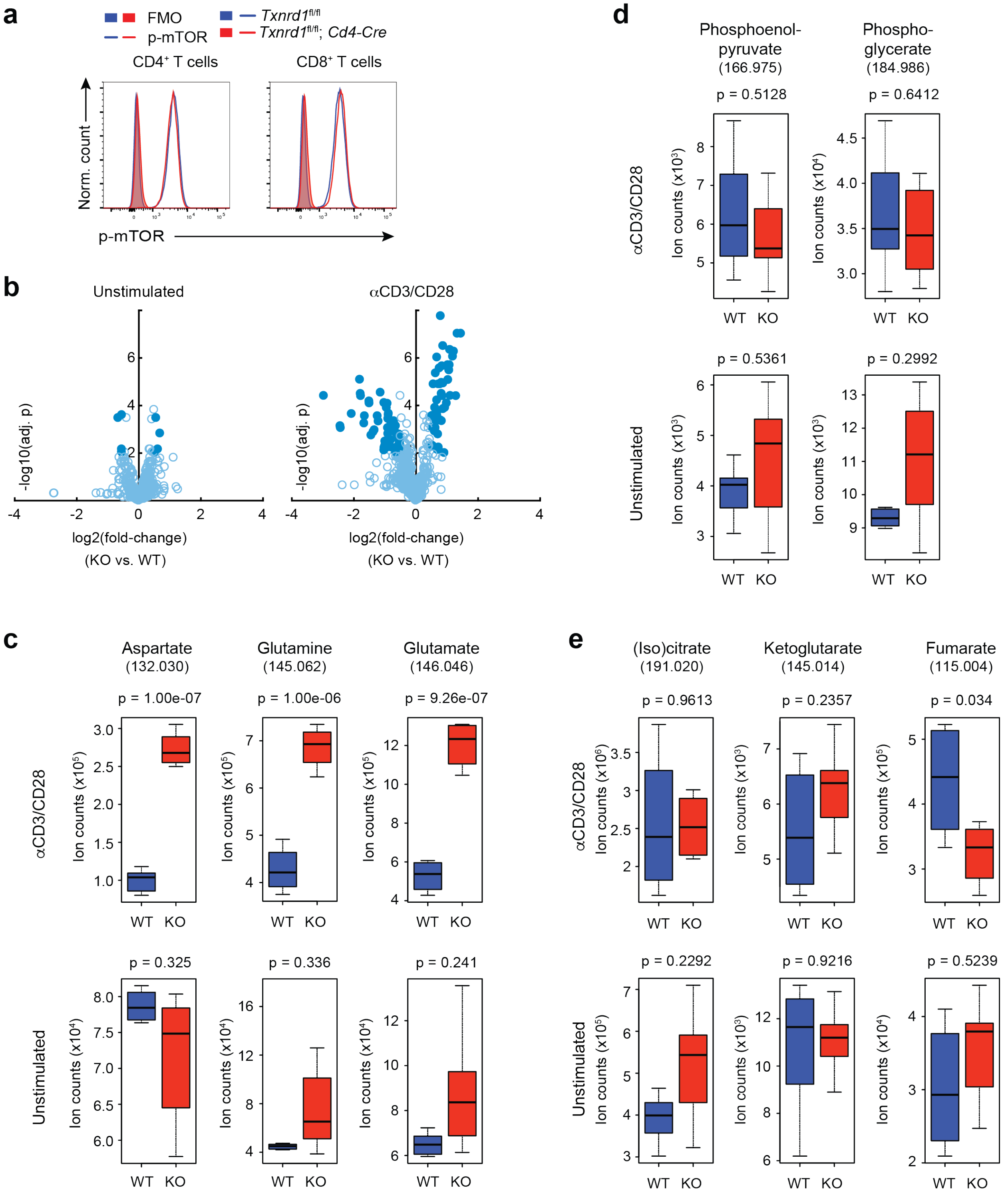
a
FMO
p-mTOR
Txnrd1fl/fl
Txnrd1fl/fl; Cd4-Cre
CD4+ T cells
CD8+ T cells
Norm. count
p-mTOR
b
Unstimulated
αCD3/CD28
-log10(adj. p)
log2(fold-change)
(KO vs. WT)
c
Aspartate
(132.030)
p = 1.00e-07
Glutamine
(145.062)
p = 1.00e-06
Glutamate
(146.046)
p = 9.26e-07
αCD3/CD28
Ion counts (x10^5)
WT KO
p = 0.325
p = 0.336
p = 0.241
Unstimulated
Ion counts (x10^4)
d
Phosphoenol-
pyruvate
(166.975)
p = 0.5128
Phospho-
glycerate
(184.986)
p = 0.6412
Ion counts (x10^3)
Ion counts (x10^4)
p = 0.5361
p = 0.2992
e
(Iso)citrate
(191.020)
p = 0.9613
Ketoglutarate
(145.014)
p = 0.2357
Fumarate
(115.004)
p = 0.034
Ion counts (x10^6)
p = 0.2292
p = 0.9216
p = 0.5239

Supplementary Figure 5. *Txnrd1* deficiency leads to aspartate and glutamine/glutamate accumulation. Magnetically-sorted T cells from spleen and peripheral LNs of *Txnrd1*$^{fl/fl}$ (WT) and *Txnrd1*$^{fl/fl}$*;Cd4-Cre* mice (KO) were stimulated in αCD3/CD28-coated plates in vitro. (**a**) p-mTOR protein expression levels measured by flow cytometry after 48 hours of T cells stimulation. (**b-e**) Metabolites were analyzed by non-targeted metabolomics (four replicates were examined, each analyzed twice; as described for Fig. 5). (**b**) Volcano plots showing comparison of identified and annotated metabolites from stimulated (and unstimulated as control) WT and KO T cells. Closed circles indicate metabolites that changed significantly ($|Log2(fold\text{-}change)| > 0.5$; $p < 0.01$). (**c-e**) Box plots displaying aspartate, glutamine and glutamate (**c**), glycolysis metabolites (**d**) and TCA cycle metabolite levels in WT and KO T cells (**e**). Numbers in brackets represent the mass-to-charge ratio of the detected ions. Data are representative of two independent experiments. P-values of volcano plots were adjusted using Benjamini-Hochberg procedure (**b**). The boxes represent the 25th to 75th percentiles, the horizontal lines within the boxes indicate the medians, and the whiskers are drawn to indicate the 1.5 x Inter Quartile Range (3rd quartile – 1st quartile; **c-e**). Student's *t* test (two-tailed, unpaired) corrected by the Benjamini-Hochberg multiple testing was used to compare WT and KO groups (**c-e**).

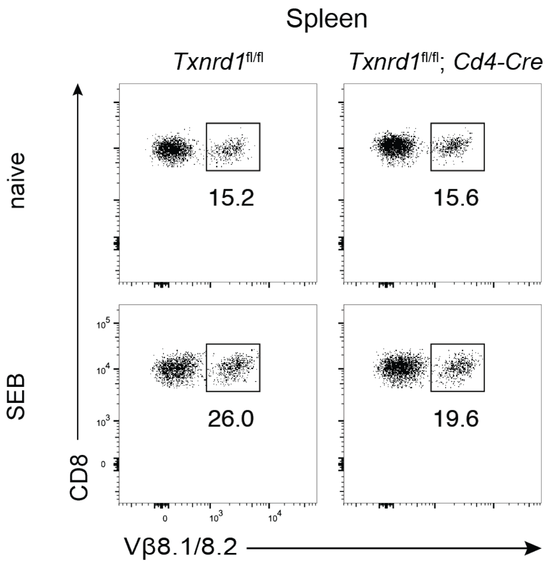

Supplementary Figure 6. Representative FACS plots for Fig. 6b. *Txnrd1^fl/fl^* and *Txnrd1^fl/fl^;Cd4-Cre* mice were treated i.p. with 100μg of SEB or PBS (naïve). Representative FACS plots showing Vβ8.1/8.2 expression of the $CD8^+$ T cells, which were FACS-sorted for RNA-seq analysis (shown in Fig. 6).

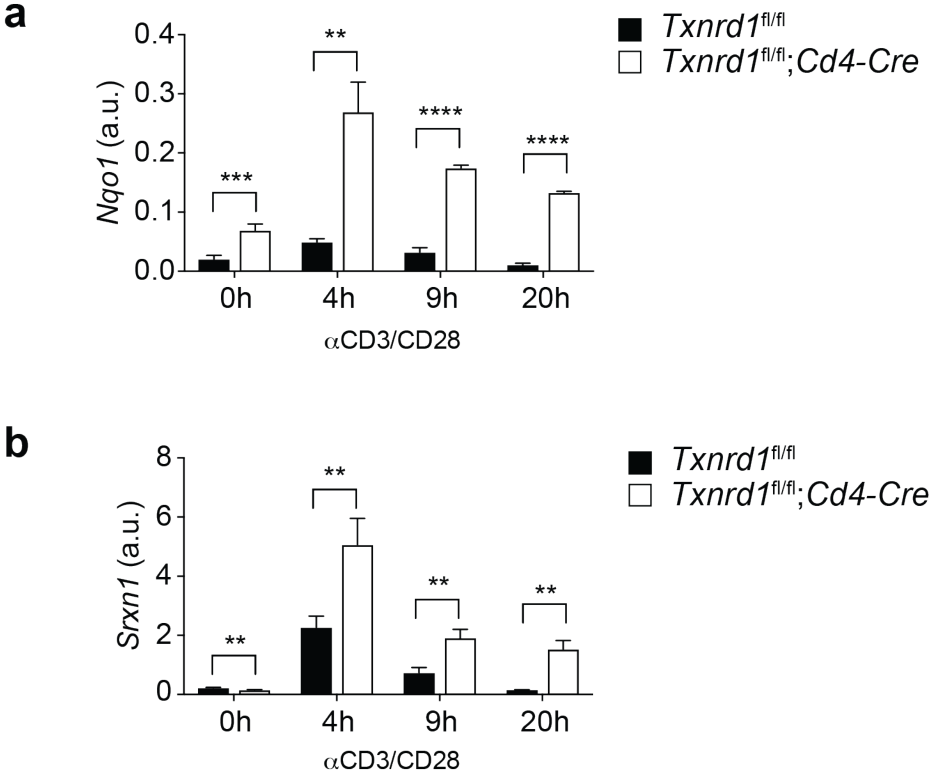

Supplementary Figure 7. Increased expression of Nrf2 target genes in *Txnrd1*-deficient T cells. (**a-b**) Magnetically-sorted T cells from spleen and peripheral LNs of naïve *Txnrd1$^{fl/fl}$* and *Txnrd1$^{fl/fl}$;Cd4-Cre* mice were stimulated in αCD3/CD28-coated plates. *Nqo1* (**a**) and *Srxn1* (**b**) expression was assessed using RT-PCR at indicated times (n=3). Bar graphs show mean + standard deviation. Data are representative of two independent experiments. Student's *t* test (two-tailed, unpaired) was used to compare *Txnrd1$^{fl/fl}$* and *Txnrd1$^{fl/fl}$;Cd4-Cre* groups: **, P ≤ 0.01; ***, P ≤ 0.001; ****, P ≤ 0.0001.

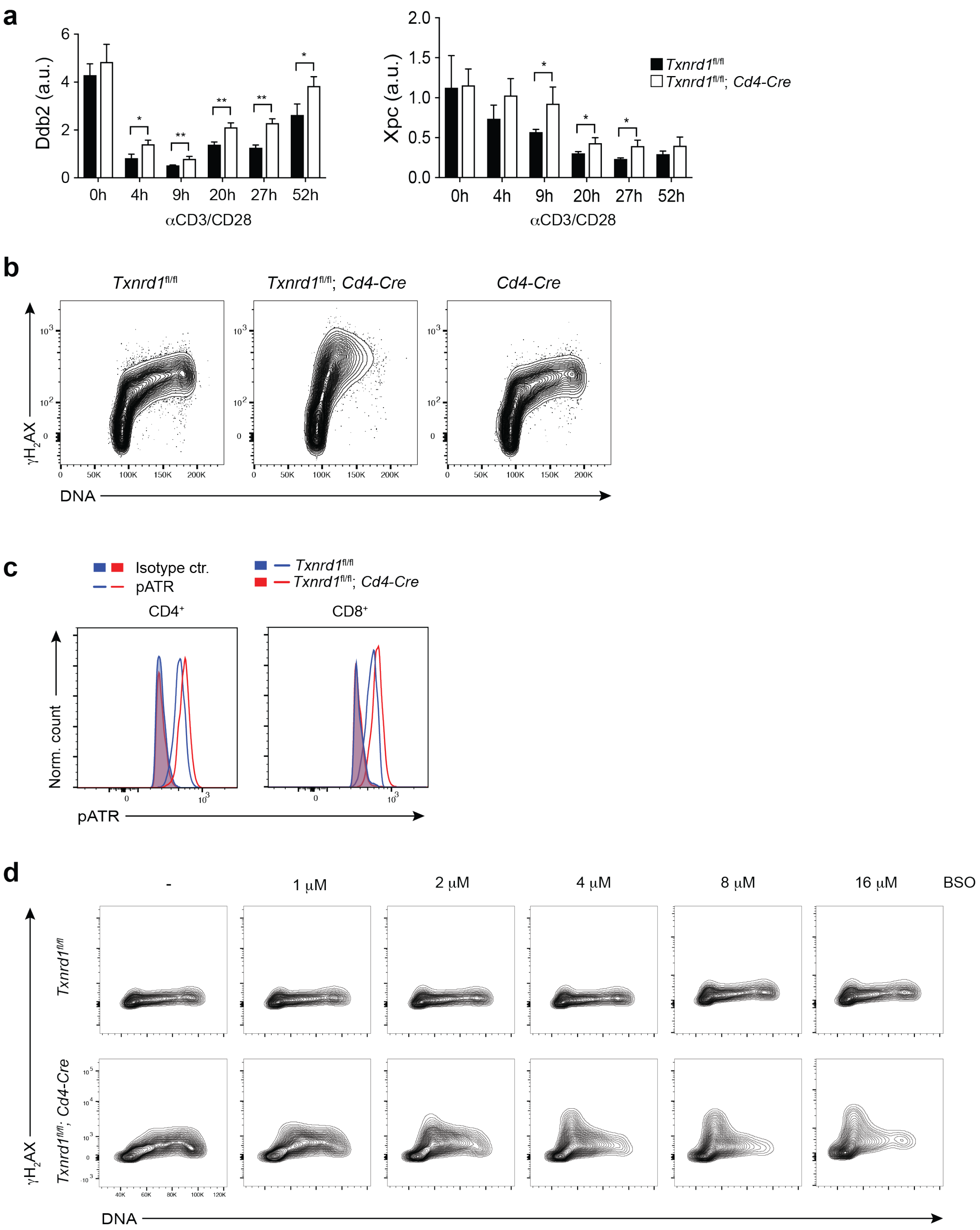

a
Ddb2 (a.u.)
0h
4h
9h
20h
27h
52h
αCD3/CD28
Xpc (a.u.)
Txnrd1fl/fl
Txnrd1fl/fl; Cd4-Cre
b
Txnrd1fl/fl
Txnrd1fl/fl; Cd4-Cre
Cd4-Cre
γH2AX
DNA
c
Isotype ctr.
pATR
Txnrd1fl/fl
Txnrd1fl/fl; Cd4-Cre
CD4+
CD8+
Norm. count
pATR
d
-
1 μM
2 μM
4 μM
8 μM
16 μM
BSO
Txnrd1fl/fl
Txnrd1fl/fl; Cd4-Cre
γH2AX
DNA

Supplementary Figure 8. *Txnrd1* deficiency leads to replication stress. Magnetically-sorted T cells from spleen and peripheral LNs of *Txnrd1*$^{fl/fl}$ and *Txnrd1*$^{fl/fl}$*;Cd4-Cre* mice were stimulated in αCD3/CD28-coated plates in vitro. (**a**) *Ddb2* (left) and *Xpc* (right) expression levels in T cells determined by RT-PCR (n=3). (**b**) Representative FACS plots depicting γH_2AX levels and DNA content in T cells from *Txnrd1*$^{fl/fl}$ (left), *Txnrd1*$^{fl/fl}$*;Cd4-Cre* (middle) and *Cd4-Cre* (right) mice after 30h of stimulation. (**c**) Representative FACS plots depicting pATR (S428) levels in T cells after 30h of stimulation (pre-gated on cells in the S-phase of the cell cycle). (**d**) Representative FACS plots depicting γH_2AX levels and DNA content in T cells stimulated for 30h in the presence of the indicated concentrations of L-Buthionine-sulfoximine (BSO). Bar graphs show mean + standard deviation (**a**). Data are representative of two independent experiments. Student's *t* test (two-tailed, unpaired) was used to compare *Txnrd1*$^{fl/fl}$ and *Txnrd1*$^{fl/fl}$*;Cd4-Cre* groups (**a**): *, P ≤ 0.05; **, P ≤ 0.01.

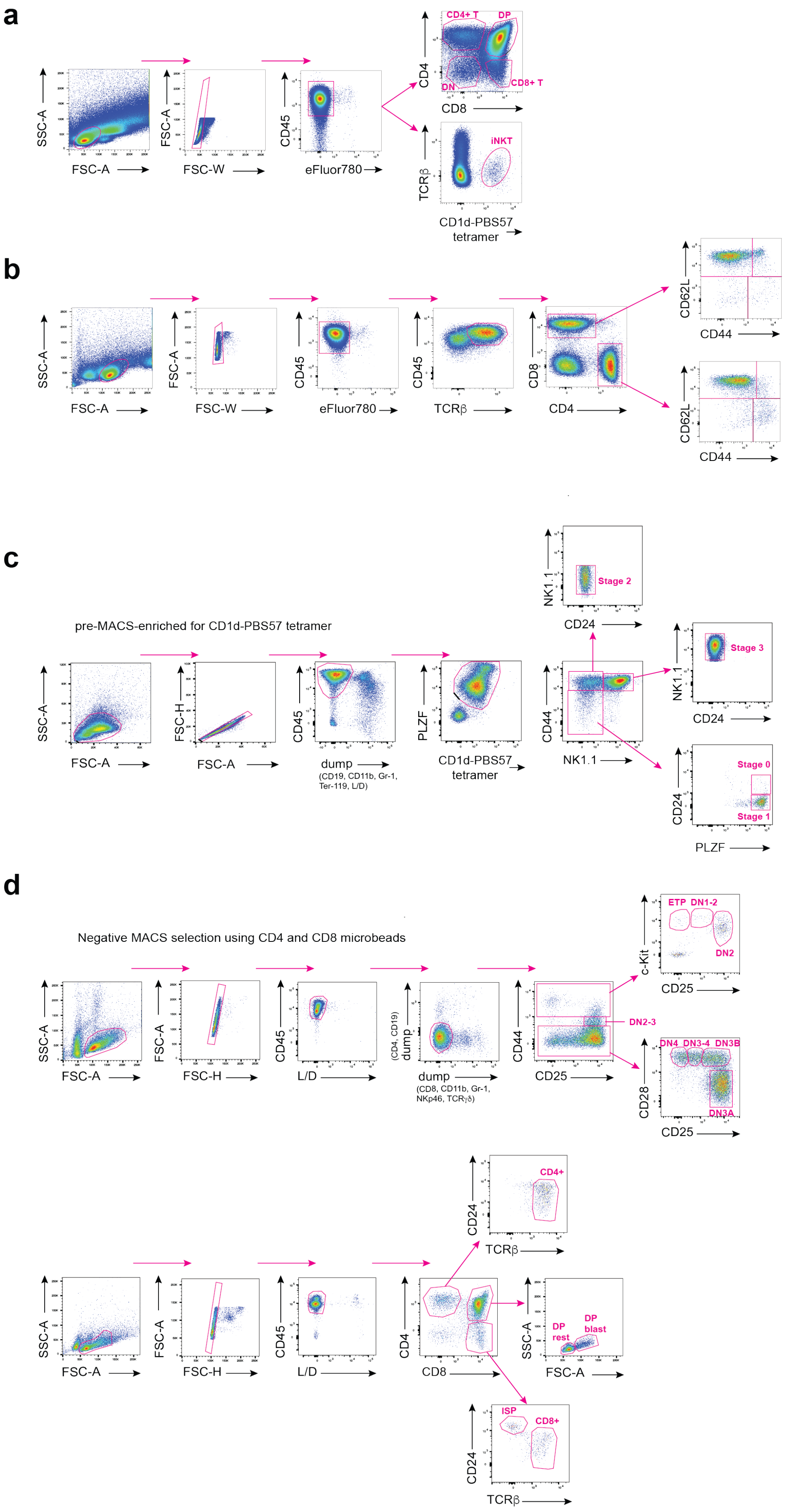
a
SSC-A
FSC-A
FSC-A
FSC-W
CD45
eFluor780
CD4+ T
DP
DN
CD8+ T
CD4
CD8
iNKT
TCRβ
CD1d-PBS57 tetramer
b
SSC-A
FSC-A
FSC-A
FSC-W
CD45
eFluor780
CD45
TCRβ
CD8
CD4
CD62L
CD44
CD62L
CD44
c
pre-MACS-enriched for CD1d-PBS57 tetramer
SSC-A
FSC-A
FSC-H
FSC-A
CD45
dump
(CD19, CD11b, Gr-1, Ter-119, L/D)
PLZF
CD1d-PBS57 tetramer
CD44
NK1.1
NK1.1
CD24
Stage 2
NK1.1
CD24
Stage 3
CD24
PLZF
Stage 0
Stage 1
d
Negative MACS selection using CD4 and CD8 microbeads
SSC-A
FSC-A
FSC-A
FSC-H
CD45
L/D
dump
(CD4, CD19)
dump
(CD8, CD11b, Gr-1, NKp46, TCRγδ)
CD44
CD25
DN2-3
c-Kit
CD25
ETP
DN1-2
DN2
CD28
CD25
DN4
DN3-4
DN3B
DN3A
SSC-A
FSC-A
FSC-A
FSC-H
CD45
L/D
CD4
CD8
CD24
TCRβ
CD4+
SSC-A
FSC-A
DP rest
DP blast
CD24
TCRβ
ISP
CD8+

Supplementary Figure 9. Gating strategies for Fig. 1 and Supplementary Fig. 1. (a-d) Representative FACS plots showing gating strategies for Fig. 1a, d, f and Supplementary Fig. 1a, b, g **(a)**; for Fig. 1b-e and Supplementary Fig. 1a-f **(b)**; for Fig. 1g **(c)**; and for Fig. 1h and Supplementary Fig. 1h **(d)**. For analysis of the different iNKT developmental stages, single thymi were MACS-enriched with APC-labeled CD1d-PBS57-tetramer and anti-APC microbeads **(c)**. Before FACS-sorting, DN thymocytes were enriched by negative selection using microbeads conjugated to anti-mouse CD4 and anti-mouse CD8 **(d; top)**.

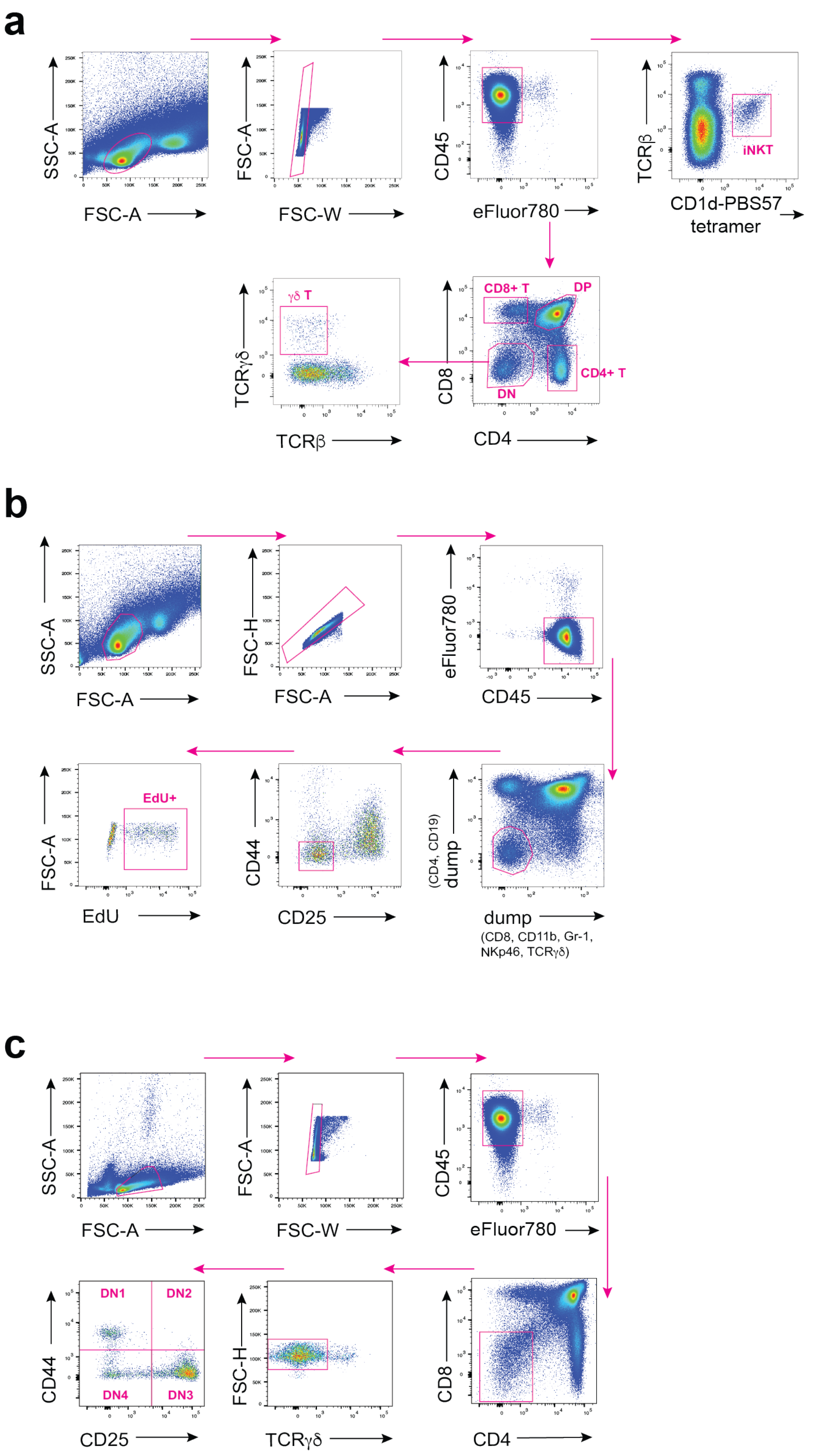
a
SSC-A
FSC-A
FSC-A
FSC-W
CD45
eFluor780
TCRβ
CD1d-PBS57 tetramer
iNKT
TCRγδ
γδ T
TCRβ
CD8
CD4
CD8+ T
DP
DN
CD4+ T
b
SSC-A
FSC-A
FSC-H
FSC-A
eFluor780
CD45
FSC-A
EdU+
EdU
CD44
CD25
(CD4, CD19)
dump
dump
(CD8, CD11b, Gr-1, NKp46, TCRγδ)
c
SSC-A
FSC-A
FSC-A
FSC-W
CD45
eFluor780
CD44
CD25
DN1
DN2
DN4
DN3
FSC-H
TCRγδ
CD8
CD4

Supplementary Figure 10. Gating strategies for Fig. 2 and Supplementary Fig. 2. (a-c) Representative FACS plots showing gating strategies for Fig. 2c, d and Supplementary Fig. 2c **(a)**; for Fig. 2e **(b)**; and for Fig. 2f and Supplementary Fig. 2d **(c)**. $CD4^+$ and $CD8^+$ T cells depicted in Supplementary Fig. 2e were gated as described in Supplementary Fig. 9b.

a

b

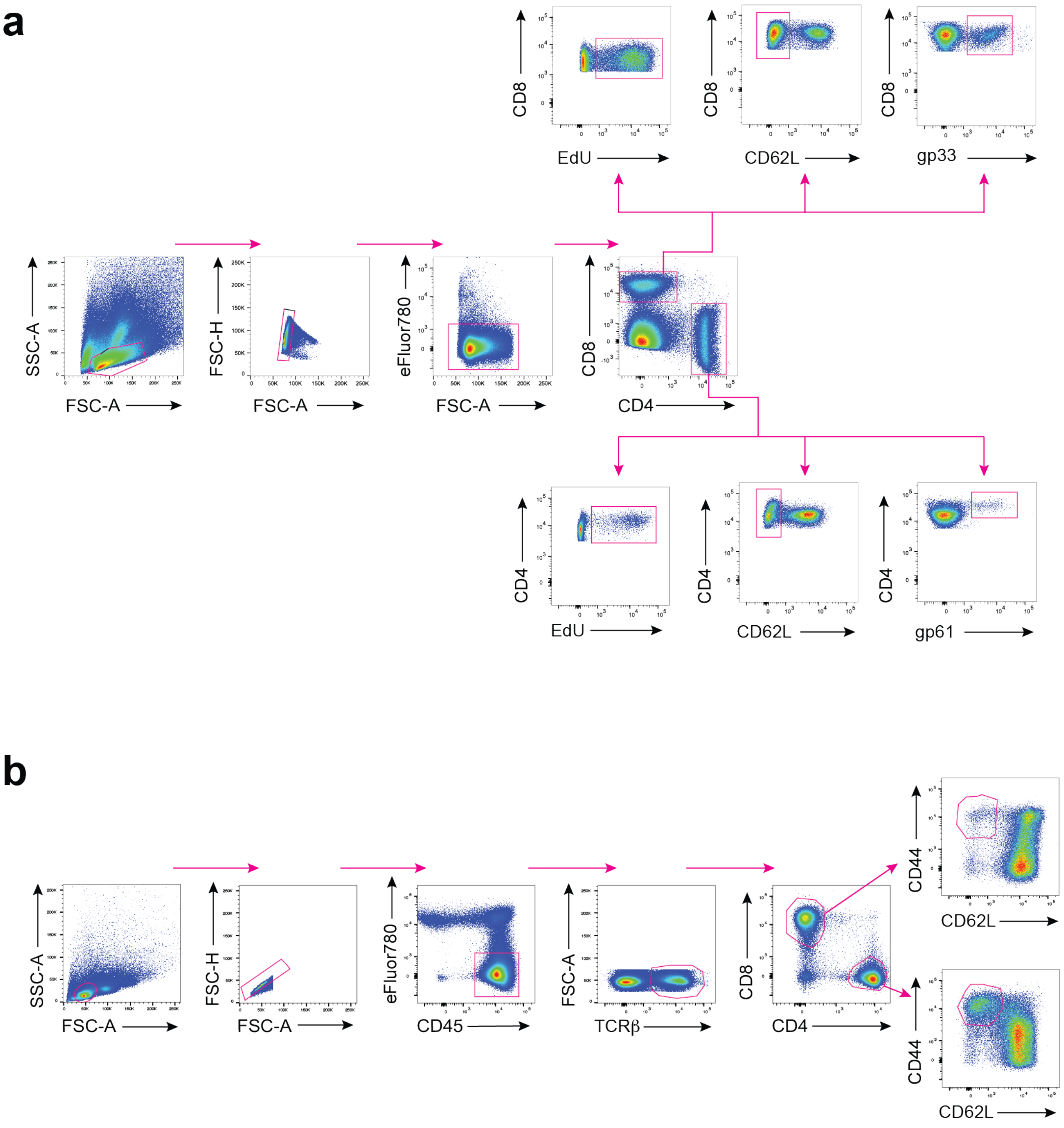

Supplementary Figure 11. Gating strategies for Fig. 3 and Supplementary Fig. 3. (a-b) Representative FACS plots showing gating strategies for Fig. 3a-c and Supplementary Fig. 3a-c **(a)**; and for Fig. 3f, g **(b)**.

Negative MACS selection using CD4 and B220 microbeads

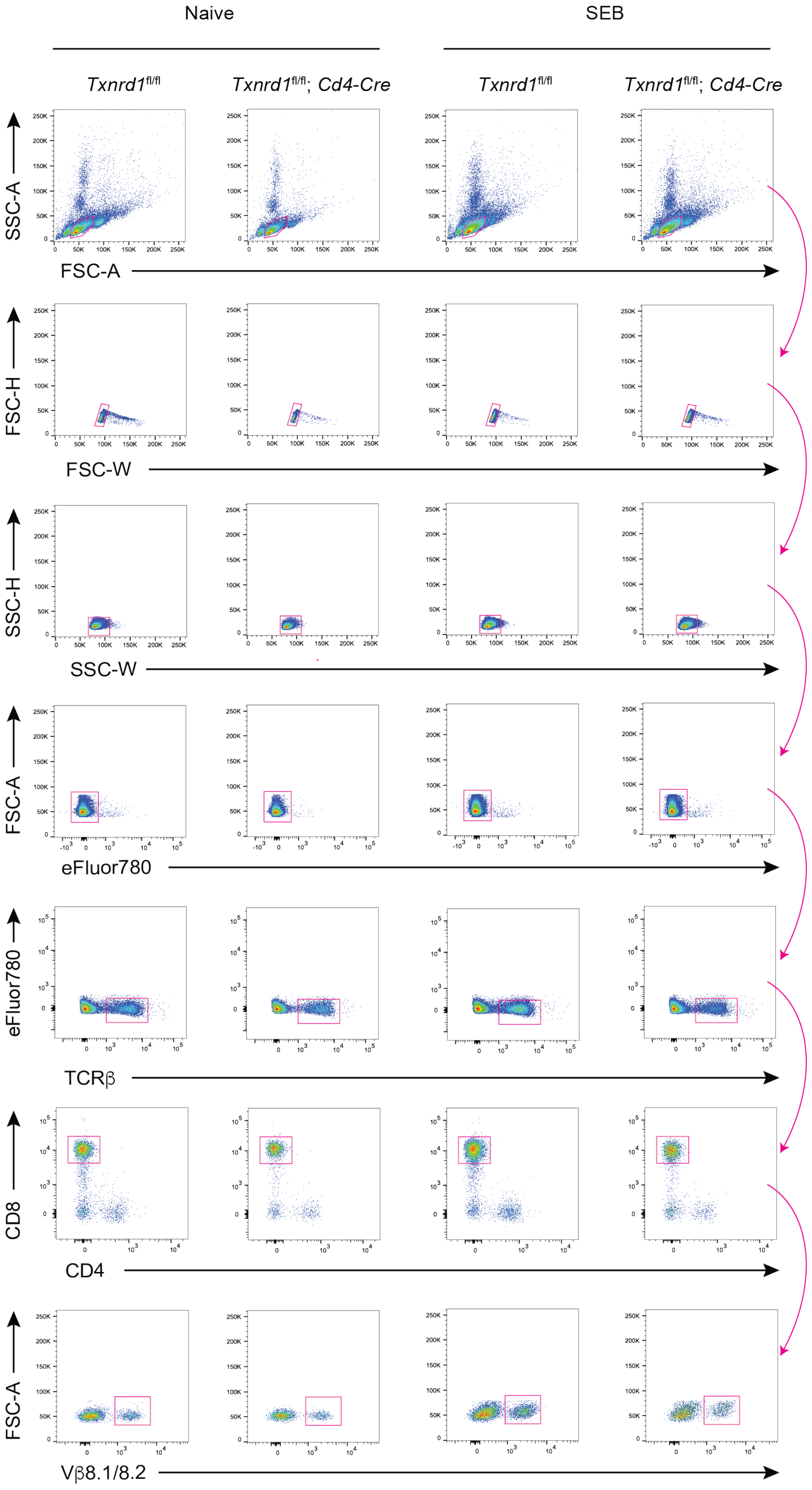

Supplementary Figure 12. Gating strategy for FACS-sorted SEB-stimulated CD8$^+$ T cells used for RNA-seq in Fig. 6. Before FACS-sorting, splenic CD8$^+$ T cells were enriched by negative selection using microbeads conjugated to anti-mouse CD4 and anti-mouse B220.

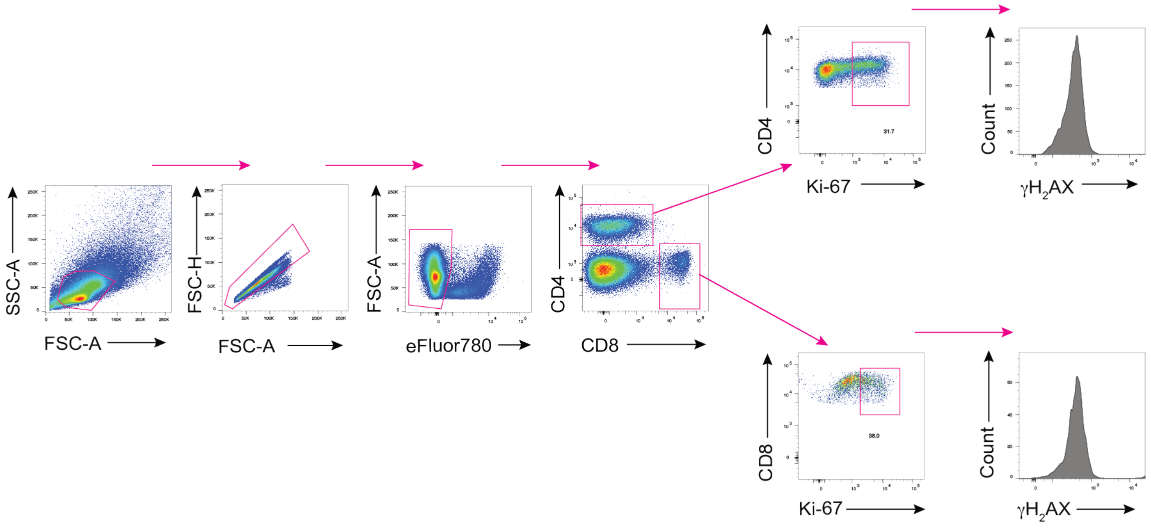

Supplementary Figure 13. Gating strategies for Fig. 7d. The first four FACS plots of the shown gating strategy were also used to gate CD4$^+$ and CD8$^+$ T cells for all the in vitro experiments.

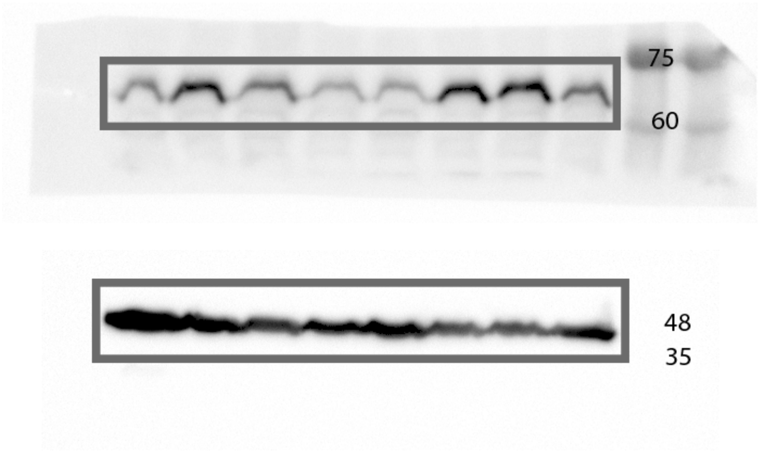

Supplementary Figure 14. Uncropped Figure 5b. A molecular weight standard (kDa) is marked on the right.

Supplementary Tables

Supplementary Table 1. Single best predictor analysis of the RNA-seq data shown in Fig. 6. Linear regression model was performed to find the single best predictors in SEB-treated groups. Genes with *standard deviation* > 2 are depicted (genes were sorted according to descending standard deviation).

Gene	Std dev	R-squared
Cdkn1a	2.750	0.918
Trav8d-2	2.670	0.996
Trav8n-2	2.635	0.967
Trav14d-3-dv8	2.584	0.935
Srxn1	2.551	0.909
Rapsn	2.417	0.993
Cxcl10	2.362	0.926
Trav10	2.333	0.979
Phf11d	2.330	0.930
Iqsec2	2.303	0.997
Ifitm1	2.286	0.958
S1pr5	2.281	0.999
Pdzd4	2.250	0.910
Trav7-4	2.153	0.908
Syne4	2.139	0.945
Palld	2.125	1.000
Trav7n-4	2.086	0.970

Supplementary Table 2. Antibodies and staining reagents for flow cytometry.

Specificity	Conjugation	Clone	Supplier	Dilution
7-AAD	-	-	eBioscience	1:50
Annexin-V (for cell death)	APC	-	BD Bioscience	1:50
B220	PerCP	RA3-6B2	Biolegend	1:400
c-Myc	-	D84C12	Cell Signalling Technology	1:100
CD117	Biotin	2B8	Biolegend	1:200
CD19	APC-Cy7	6D5	Biolegend	1:800
CD19	PE	eBio1D3	eBioscience	1:500
CD23	PE	B3B4	eBioscience	1:300
CD25	PE	PC61	eBioscience	1:400
CD28	PE	E18	eBioscience	1:300
CD3e	PE	145-2C11	eBioscience	1:300
CD4	APC	GK1.5	BD Bioscience	1:1000
CD4	APC-Cy7	GK1.5	Biolegend	1:1000
CD4	FITC	GK1.5	eBioscience	1:800
CD4	PE	GK1.5	BD Bioscience	1:500
CD4	PE-Cy7	GK1.5	Biolegend	1:1000
CD4	PerCP-Cy5.5	RM4-5	Biolegend	1:500
CD11b	PE	M1/70	BD Bioscience	1:1000
CD44	Biotin	IM7	Biolegend	1:2000
CD44	PE	IM7	BD Bioscience	1:1000
CD44	PerCP-Cy5.5	IM7	eBioscience	1:500
CD45.1	APC	A20	Biolegend	1:100
CD45.1	Biotin	A20	BD Bioscience	1:200
CD45.2	APC	104	eBioscience	1:100
CD45.2	FITC	104	eBioscience	1:300
CD45.2	PE	104	eBioscience	1:200
CD45.2	PE-Cy7	104	Biolegend	1:200
CD62L	APC	MEL-14	Biolegend	1:200
CD62L	FITC	MEL-14	eBioscience	1:500
CD62L	PE	MEL-14	BD Bioscience	1:500
CD69	FITC	H1.2F3	Biolegend	1:300
CD71	FITC	R17217	eBioscience	1:200
CD8	APC	53-6.7	eBioscience	1:600
CD8	FITC	53-6.7	eBioscience	1:600
CD8	PE	53-6.7	Biolegend	1:2000
CD8	PE-Cy7	53-6.7	Biolegend	1:1000
CD8	PerCP	53-6.7	Biolegend	1:600
eFluor® 780	-	-	eBioscience	1:2000
Fc Block CD16/32	-	2.4G2	Home-made	1:1000
FxCycle Violet Stain	-	-	Thermo Scientific	1:400
Gr-1	PE	RB6-8C5	Biolegend	1:800
Ki-67	PE	16A8	Biolegend	1:1000
LIVE/DEAD® fixable-yellow-dead-cell-stain	-	-	Life Technology	1:500
NK1.1	PE	PK136	eBioscience	1:300
Phospho-ATR (Ser428)	-	-	Cell Signalling Technology	1:100
Phospho-H_2AX (Ser139)	AF488	N1-431	BD Bioscience	1:400
Phospho-mTOR (Ser2448)	PE	MRRBY	eBioscience	1:100
PLZF	PE	9E12	Biolegend	1:1000
Rabbit-IgG(H+L)	FITC	-	SouthernBiotech	1:500
Streptavidin	APC	-	eBioscience	1:1000
Streptavidin	APC-Cy7	-	Biolegend	1:1000
Streptavidin	BV711	-	BD Bioscience	1:1000
Streptavidin	PE-Cy7	-	Biolegend	1:1000
Streptavidin	PerCP	-	Biolegend	1:500
TCRβ	APC	H57-597	Biolegend	1:500
TCRβ	PE-Cy7	H57-597	BD Bioscience	1:1000
TCR$\gamma\delta$	Biotin	GL3	BD Bioscience	1:300
Ter-119	PE	TER-119	eBioscience	1:100
Vb8.1/8.2	FITC	KJ16-133	eBioscience	1:50

Supplementary Table 3. Sequences of primers used for RT-PCR.

Target gene	Primer sequences
Cdkn1a	Forward: 5’-AGACCAGCCTGACAGATTTC-3’<br>Reverse: 5’-CTGACCCACAGCAGAAGAG-3’
c-Myc	Forward: 5’-TTGAAGGCTGGATTTCCTTTGGGC-3’<br>Reverse: 5’-TCGTCGCAGATGAAATAGGGCTGT-3’
Ddb2	Forward: 5’-GGCAAGGAGCTTTGGAAC-3’<br>Reverse: 5’-GTTTGATCTATGGAGGCTGTG-3’
Grx1	Forward: 5’-TGCAGAAAGACCCAAGAAATCCTCAGTCA-3’<br>Reverse: 5’-TGGAGATTAGATCACTGCATCCGCCTATG-3’
Nqo1	Forward: 5’-GGTGATATTTCAGTTCCCATTG-3’<br>Reverse: 5’-ACTCTCTCAAACCAGCCTTTC-3’
Srxn1	Forward: 5’-GGTGGACACGATCCTGGCGG-3’<br>Reverse: 5’-GGTAGGCTGCATAGCGGTGGC-3’
Txn1	Forward: 5’-ATGACTGCCAGGATGTTGC-3’<br>Reverse: 5’-CCTTGTTAGCACCGGAGAAC-3’
Txnip	Forward: 5’-CCTGACCTAATGGCACCAG-3’<br>Reverse: 5’-AGGAATGAACATGCAGGAAAC-3’
Txnrd1	Forward: 5’-AAAGACGATGAACGTGTCG-3’<br>Reverse: 5’-CTTAGTCAGCCCACACTTGAG-3’
Txnrd1 DNA	Forward: 5’-ACAGGAGTGATCCCCACAGACC-3’<br>Reverse: 5’-CTGGAACCGCCCTGAATATCACC-3’
Txnrd1 mRNA in KO	Forward: 5’-GCTGACTAAGCAGCAGCTGG-3’<br>Reverse: 5’-AACCTCAGCAGCCAGACTGG-3’
Xpc	Forward: 5’-GCAAGAAGGCAAAGAAACAC-3’<br>Reverse: 5’-TTCAGTAAGCTCTTCCACCTC-3’
Tbp<br>(housekeeping for mRNA)	Forward: 5’-TTGACCTAAAGACCATTGCACTTC-3’<br>Reverse: 5’-TTCTCATGATGACTGCAGCAAA-3’
Txnrd1<br>(housekeeping for DNA)	Forward: 5’-ACAGATCGAAGCAGGAACAC-3’<br>Reverse: 5’-TTCAGAGAGGAAAGTCACCC-3’

Supplementary Table 4. MRM settings used for targeted metabolomics.

Metabolite	Q1	Q2
AMP	346	134
GMP	362	79
dATP	490	159
dGTP	506	79
