# Supplementary material for: The thioredoxin-1 system is essential for fueling DNA synthesis during T-cell metabolic reprogramming and proliferation
Source: Nat Commun. 2018 May 10;9:1851. doi: 10.1038/s41467-018-04274-w (PMC5945637; doi:10.1038/s41467-018-04274-w)
Supplement: Supplementary file 3 — Description of Additional Supplementary Files [file 41467_2018_4274_MOESM3_ESM.pdf]

Description of Additional Supplementary Files

File Name: Supplementary Data 1
Description: **Metabolite abundances in *Txnrd1*-sufficient and -deficient T cells**
